# The association between introduction of the micro-axial flow pump Impella in hospitals and in-hospital mortality in patients treated with extracorporeal membrane oxygenation: interrupted time-series analyses

**DOI:** 10.1186/s13613-024-01381-4

**Published:** 2024-09-28

**Authors:** Jun Nakata, Hiroyuki Ohbe, Toru Takiguchi, Yuji Nishimoto, Mikio Nakajima, Yusuke Sasabuchi, Toshiaki Isogai, Hiroki Matsui, Takeshi Yamamoto, Shoji Yokobori, Kuniya Asai, Hideo Yasunaga

**Affiliations:** 1 Division of Cardiovascular Intensive Care, Nippon Medical School, Tokyo, Japan; 2 Department of Clinical Epidemiology and Health Economics, School of Public Health, The University of Tokyo, 7-3-1 Hongo, Bunkyo-Ku, Tokyo, 1130033 Japan; 3 Department of Emergency and Critical Care Medicine, Nippon Medical School, Tokyo, Japan; 4 Division of Cardiology, Osaka General Medical Center, Osaka, Japan; 5 Emergency and Critical Care Center, Tokyo Metropolitan Hiroo Hospital, Tokyo, Japan; 6 Department of Real-World Evidence, Graduate School of Medicine, The University of Tokyo, Tokyo, Japan; 7 Department of Cardiology, Tokyo Metropolitan Tama Medical Center, Tokyo, Japan

**Keywords:** Micro-axial flow pump, Impella, Venoarterial extracorporeal membrane oxygenation, Interrupted time series analysis, Mortality, Cost

## Abstract

**Background:**

The micro-axial flow pump Impella, a new mechanical circulatory device for cardiogenic shock, is still only available in a limited number of hospitals, due to the facility certification requirements and insufficient evidence of the benefit of introducing Impella in hospitals. This study aimed to evaluate the impact of introducing Impella in hospitals on in-hospital mortality of patients treated with extracorporeal membrane oxygenation (ECMO).

**Methods:**

Using a nationwide Japanese inpatient database, we identified patients who received ECMO during hospitalization between 1 April 2014 and 31 March 2021. A hospital-level propensity score–matched cohort was created matching hospitals that introduced Impella (exposure group) to those that did not introduce Impella (control group). The inclusion period in each hospital was divided into two time periods according to the time of Impella introduction in the exposure group and the corresponding hospital in the control group (before and after exposure). The primary outcome was in-hospital mortality. Uncontrolled and controlled interrupted time-series analyses involved before–after exposure comparison and exposure–control comparison.

**Results:**

Out of 34,379 eligible patients, we created a matched cohort of 8351 patients from 86 hospitals with Impella introduction (exposure group) and 7230 patients from 86 hospitals without Impella introduction (control group). In-hospital mortality before and after exposure was 62.5% and 59.3, respectively, in the exposure group; and 66.8% and 63.7%, respectively, in the control group. Uncontrolled interrupted time-series analysis showed no significant level change or trend change in the before–after exposure comparison in both the exposure and the control groups. Controlled interrupted time-series analysis also showed no significant level change (−0.01%; 95% confidence intervals −5.36% to + 5.33%) or trend change (+ 0.10%, −0.30% to + 0.40%) after exposure in the exposure–control comparison.

**Conclusions:**

This nationwide inpatient database study showed no association between Impella introduction in hospitals and in-hospital mortality of patients who underwent ECMO. Because this study confined itself to analze of the impact of the introduction of Impella solely at the hospital level, further detailed studies are warranted to assess its efficacy at the patient level.

**Supplementary Information:**

The online version contains supplementary material available at 10.1186/s13613-024-01381-4.

## Introduction

Venoarterial extracorporeal membrane oxygenation (ECMO) is increasingly being used for circulatory support in patients with refractory cardiogenic shock and cardiac arrest [1]. Although ECMO has the advantage of elevating blood pressure, it may potentially increase left ventricular (LV) afterload, inducing pulmonary oedema, LV thrombus, and myocardial wall stress [2]. Recently, the micro-axial flow pump called the “Impella”, has been developed. The device is designed to complement the shortcomings of ECMO by pumping blood from the LV into the ascending aorta [3].

Previous studies showed that ECMO treatment in combination with Impella was associated with improved short-term mortality compared with ECMO treatment alone [4–7], and guidelines recommended comprehensive management using temporary mechanical circulatory support (t-MCS) including ECMO and Impella for cardiogenic shock [8, 9]. In Japan, certified facilities have begun to use Impella combined with ECMO to improve the outcome of cardiogenic shock since 02 October 2017. However, Impella is currently available in only a limited number of hospitals in Japan due to the regulatory requirements for facilities and staffing structure as well as implementation costs. Many facilities are faced with the decision on whether they should introduce Impella. Although the effect of Impella has been examined at the patient level, the association between introducing Impella in hospitals and the clinical outcomes of patients treated with ECMO and the associated costs thereof remains unclear.

Therefore, the present study aimed to evaluate the impact of Impella introduction in hospitals on clinical outcomes and healthcare costs, using a nationwide inpatient administrative database.

## Methods

### Study design

We conducted uncontrolled and controlled interrupted time-series analyses involving comparisons of hospitals; before vs after Impella introduction and with vs without Impella introduction, focusing on patients treated with ECMO [10]. To select an appropriate control group that would be as similar as possible to the exposure group, we conducted hospital-level propensity score matching.

### Data source

We used the Japanese Diagnosis Procedure Combination inpatient database, which contained administrative claims data and discharge abstracts from more than 1,500 acute care hospitals and covered approximately 90% of all tertiary emergency hospitals in Japan [11]. The database includes the following patient-level data for all hospitalizations: age, sex, diagnoses (main diagnosis, admission-precipitating diagnosis, most resource-consuming diagnosis, second-most resource-consuming diagnosis, comorbidities present on admission, and complications arising after admission) recorded according to the International Classification of Diseases 10th Revision (ICD-10) codes; daily procedures recorded according to Japanese medical procedure codes; daily drug administration; and admission and discharge status [11]. ICD-10 codes for cardiovascular diseases are shown in Table S1 (see Supplementary Materials). A previous validation study showed that the specificity of the recorded diagnoses in the database exceeded 96%, the sensitivity of the diagnoses ranged from 50 to 80%, and the specificity and sensitivity of procedures both exceeded 90% [12].

### Study population

From the database, we identified all patients aged ≥ 18 years who received ECMO during hospitalization between 1 April 2014 and 31 March 2021. We excluded patients admitted to hospitals that did not have at least one patient treated with ECMO in the database in eight consecutive years during the period from April 2014 to March 2021.

### Outcomes

The primary outcome was in-hospital mortality. The secondary outcomes were length of hospital stay, duration of ECMO, total hospitalization costs, and complications (major bleeding, ischemic stroke, and both). Total hospitalization costs were estimated based on reference prices in the Japanese national fee schedule that determine item-by-item prices for all inpatient services [13], and were converted to United States dollars at the rate of 110 Japanese yen to the dollar. Major bleeding was defined as the presence of either intracranial bleeding (ICD-10 code: I61), intraspinal bleeding (G951), pericardial haematomas (I312), intra-abdominal or retroperitoneal haematomas (K661), intra-articular bleeding (M250), intraocular bleeding (H448), or compartment syndrome (M622), in accordance with the definitions of International Society of Thrombosis and Haemostasis [14]. All patients were followed until death or discharge from the hospital.

### Impella in Japan

Impella was approved for reimbursement under national health insurance in Japan on 01 September 2017, but the timing of Impella introduction varied from hospital to hospital. All hospitals that have introduced Impella are facilities certified by the academic-based Japan Impella Committee [15]. A certified facility must satisfy the following criteria. (1) The facility must have emergency/intensive care unit with sufficient experience in cardiogenic shock treatment. (2) The facility must have qualified cardiology specialists and cardiovascular surgery specialists (paediatric hospitals must have qualified paediatric cardiology specialists). In addition, a Heart Team consisting of intensivist, certified interventional cardiologists, etc. must be in place for the circulatory assist therapy. (3) The facility must have three or more certified extracorporeal circulation technicians or certified artificial organ management technicians. (4) The annual number of cardiovascular surgeries must be 100 or more (for paediatric hospitals, the annual number of cardiac surgeries for patients aged under 18 years must be 50 or more). (5) The facility must have handled more than 30 intra-aortic balloon pumping (IABP) cases and more than 20 percutaneous cardio-pulmonary support (PCPS)/ECMO cases are required in the last 3 years. (6) Finally, the facility must have handled more than 300 cases of percutaneous coronary intervention within the last 3 years. Guidelines for the appropriate use of Impella have been published by the academic-based Japan Impella Committee [15], and the indication for Impella is defined as drug-resistant acute heart failure (such as cardiogenic shock) for which haemodynamic support by existing circulatory assist devices (such as IABP and PCPS) is expected to be insufficient.

### Definitions of before–after exposure period and exposure–control groups

Exposure was defined as the introduction of Impella in a hospital. The exposure group was defined as patients admitted to hospitals where Impella had been used at least once during the study period. The control group was defined as patients admitted to hospitals where Impella was never used during the study period. For before–after comparison, the study period was divided into before and after the exposure date (defined as the date of the first use of Impella at the hospital during the study period). In the control group, the exposure date for the before–after comparison was defined as the exposure date of the hospital in the exposure group which was matched to the hospital in the control group by hospital-level propensity score matching (details are described below).

### Hospital-level propensity score matching

Before applying the uncontrolled and controlled interrupted time-series analyses, we performed hospital-level propensity score matching to balance the hospital characteristics between the exposure and control groups. We first employed hospital- and year-level logistic regression models using teaching hospital, tertiary emergency hospital, and annual hospital volume of ECMO as covariates to compute the propensity scores for hospitals with Impella introduction. Next, for each fiscal year, we created a cohort of hospitals in the exposure group that introduced Impella at that year and a control group that had not previously been matched with the exposure group. Then, using the created cohort for each fiscal year, we performed one-to-one nearest-neighbour matching without replacement using the estimated propensity scores, setting a calliper width at 20% of the standard deviation of the propensity scores [16]. After hospital-level propensity score matching, we excluded patients who received ECMO more than 54 months before or more than 30 months after the date of exposure.

### Uncontrolled and controlled interrupted time-series analyses

All analyses described below were performed at patient level in the hospital-level matched cohort. We checked the balance of the characteristics before and after exposure in the exposure and control groups. We also examined the balance between the exposure and control groups. The characteristics were compared using standardized mean differences. An absolute standardized mean difference ≤ 10% denotes a negligible imbalance between the two groups [17].Change in outcome before and after exposure was evaluated using segmented linear regression with interrupted time-series [18]. First, uncontrolled interrupted time-series analyses were done for the exposure and control groups, separately. The equation for the uncontrolled interrupted time-series analysis is as follows:

$$Y_{t} = \beta_{0} + \beta_{1} T + \beta_{2} X_{t} + \beta_{3} TX_{t}$$

where $Y_{t}$ is the outcome, $T$ is the month since the beginning of exposure, and $X_{t}$ is a dummy variable indicating before or after the exposure. In this model, ${\beta }_{0}$ represents the baseline level of the outcome at the beginning of the study period, ${\beta }_{1}$ represents the baseline trend before Impella introduction,$\beta_{2}$ represents the level change immediately after Impella introduction, and $\beta_{3}$ represents the trend change after Impella introduction compared to the baseline trend.

Next, a controlled interrupted time-series analysis was performed, incorporating exposure and control. The equation in the controlled interrupted time-series analysis is as follows [19]:

$$Y_{t} = \beta_{0} + \beta_{1} T + \beta_{2} X_{t} + \beta_{3} TX_{t} + \beta_{4} G + \beta_{5} GT + \beta_{6} GX_{t} + \beta_{7} GX_{t} T$$

where $G$ represents the exposure group $(G = 1)$ or control group ($G=0$). In this model, ${\beta }_{4}$ represents the difference in baseline level of the outcome at the beginning of the study period in the exposure–control comparison, ${\beta }_{5}$ represents the trend change before pVAD introduction in the exposure–control comparison, ${\beta }_{6}$ represents the level change associated with Impella introduction in the exposure–control comparison, and ${\beta }_{7}$ represents the trend change associated with Impella introduction in the exposure–control comparison.

Categorical variables are expressed as count and percentage, and continuous variables as mean and standard deviations (SD) or median and interquartile range. All analyses were performed using Stata/SE 17.0 software (StataCorp, College Station, TX, USA). All reported P-values were two-sided, and P-values < 0.05 were considered statistically significant.

## Results

**Fig. 1:**
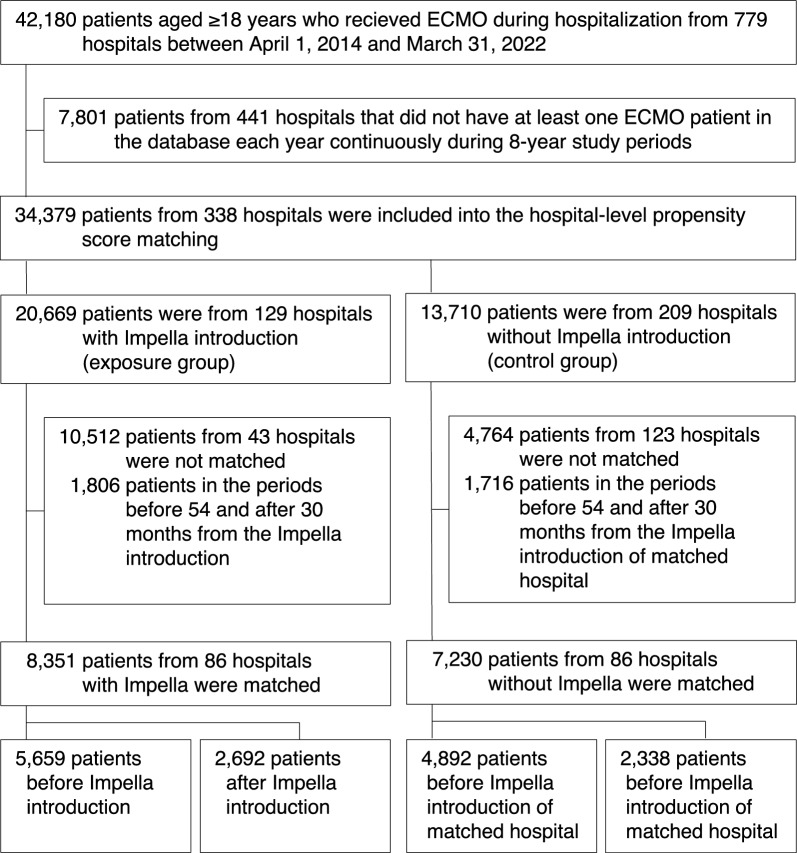
Patient selection flowchart. ECMO, extracorporeal membrane oxygenation; Impella

During the study period, we identified 34,379 eligible patients who received ECMO from 338 hospitals (Fig. 1). Of these, 20,669 patients were from 129 hospitals with Impella introduction and 13,710 patients were from 209 hospitals without Impella introduction. After hospital-level propensity score matching, 8351 patients from 86 hospitals with Impella (exposure group) and 7230 patients from 86 hospitals without Impella (control group) were matched and included in the uncontrolled and controlled interrupted time-series analyses. The hospital characteristics before and after matching are shown in Table S2 (see Supplementary Materials).

Table S3 (see Supplementary Materials) shows comparisons of the characteristics and outcomes between the exposure and control groups of the hospital-level propensity score–matched cohort. Compared with patients in the control group, patients in the exposure group were more likely to be admitted to hospitals with a high volume of ECMO, have a better consciousness level at admission, have independent physical function at admission, be transferred from other hospitals, have more valvular and aortic diseases, receive a surgical or percutaneous valvular procedure, and receive a blood transfusion and catecholamines. In contrast, compared with patients in the exposure group, those in the control group were more likely to be admitted to a tertiary emergency hospital, use an ambulance, have a cardiac arrest, and receive extracorporeal cardio-pulmonary resuscitation. The crude outcomes of the exposure and control groups are shown in Table S4 (see Supplementary Materials)**.**

Comparisons of the patient characteristics of the matched cohort between before and after exposure in the exposure and control groups are shown in Table 1. In the exposure group, ECMO with IABP was used in 57.7% (3263/5659 patients) before and 36.7% (988/2692 patients) after exposure. ECMO with Impella was used in 14.3% (384/2692 patients) after exposure, and the median annual number of cases that underwent ECMO with Impella was 5 (interquartile range 2–8, lowest 1 and highest 19) per hospital in the exposure group. In the control group, ECMO with IABP was used in 50.3% (2462/4892 patients) before and 48.1% (1125/2338 patients) after exposure. Most of the patients and hospital characteristics were comparable before and after exposure in both the exposure and control groups. Comparisons of the patient characteristics and outcomes between patients receiving ECMO with and without an Impella device in hospitals employing Impella devices after exposure are shown in Table S5 (see Supplementary Materials).

**Table 1:** Patient characteristics comparison before and after the introduction of Impella in hospitals with and without Impella introduction

Variables	Hospitals with impella			Hospitals without Impella		
	Before	After		Before	After	
	(*n* = 5659)	(*n* = 2692)	SMD	(*n* = 4892)	(*n* = 2338)	SMD
ECMO with Impella, n (%)	0 (0.0)	384 (14.3)	56	0 (0.0)	0 (0.0)	0
ECMO with IABP, n (%)	3263 (57.7)	988 (36.7)	−43	2462 (50.3)	1125 (48.1)	−5
Fiscal year at ECMO initiation, n (%)						
2014	297 (5.2)	0 (0.0)	−33	296 (6.1)	0 (0.0)	−36
2015	716 (12.7)	0 (0.0)	−54	573 (11.7)	0 (0.0)	−52
2016	1053 (18.6)	0 (0.0)	−68	907 (18.5)	0 (0.0)	−67
2017	1368 (24.2)	20 (0.7)	−76	1,077 (22.0)	7 (0.3)	−73
2018	1153 (20.4)	198 (7.4)	−38	995 (20.3)	227 (9.7)	−30
2019	687 (12.1)	695 (25.8)	35	647 (13.2)	582 (24.9)	30
2020	305 (5.4)	889 (33.0)	75	309 (6.3)	814 (34.8)	75
2021	80 (1.4)	890 (33.1)	92	88 (1.8)	708 (30.3)	84
Hospital characteristics						
Teaching hospital, n (%)	5625 (99.4)	2684 (99.7)	5	4892 (100.0)	2338 (100.0)	
Tertiary emergency hospital, n (%)	4147 (73.3)	1915 (71.1)	−5	4065 (83.1)	1902 (81.4)	−5
Annual hospital volume of ECMO, mean (SD)	60.7 (28.0)	63.3 (24.3)	10	44.9 (20.6)	45.0 (20.9)	0
Age, years, mean (SD)	64.4 (15.5)	64.4 (15.4)	0	64.4 (14.7)	64.8 (14.2)	2
Men, *n* (%)	3985 (70.4)	1844 (68.5)	−4	3506 (71.7)	1777 (76.0)	10
Smoking history, n (%)						
Nonsmoker	2350 (41.5)	1133 (42.1)	1	2115 (43.2)	962 (41.1)	−4
Current/past smoker	1961 (34.7)	865 (32.1)	−5	1479 (30.2)	781 (33.4)	7
Unknown	1348 (23.8)	694 (25.8)	5	1298 (26.5)	595 (25.4)	−3
Body mass index at admission, kg/m^2^, n (%)						
< 18.5	493 (8.7)	226 (8.4)	−1	318 (6.5)	154 (6.6)	0
18.5–24.9	2791 (49.3)	1243 (46.2)	−6	2240 (45.8)	1057 (45.2)	−1
25.0–29.9	1215 (21.5)	599 (22.3)	2	1034 (21.1)	515 (22.0)	2
≥ 30.0	404 (7.1)	252 (9.4)	8	360 (7.4)	200 (8.6)	4
Missing data	756 (13.4)	372 (13.8)	1	940 (19.2)	412 (17.6)	−4
Japan Coma Scale at admission, *n* (%)						
0 (alert)	3120 (55.1)	1479 (54.9)	0	2008 (41.0)	1011 (43.2)	4
1–3 (dizzy)	481 (8.5)	256 (9.5)	4	382 (7.8)	178 (7.6)	−1
10–30 (somnolent)	238 (4.2)	129 (4.8)	3	192 (3.9)	86 (3.7)	−1
100–300 (coma)	1820 (32.2)	828 (30.8)	−3	2310 (47.2)	1063 (45.5)	−3
Charlson comorbidity index score, mean (SD)	1.1 (1.3)	1.1 (1.3)	−4	1.0 (1.3)	1.0 (1.2)	−1
Comorbidity of peripheral vascular diseases, n (%)	293 (5.2)	127 (4.7)	−2	208 (4.3)	57 (2.4)	−10
Physical function at admission, n (%)						
Total/severe dependence (Barthel index 0–60)	3157 (55.8)	1499 (55.7)	0	3040 (62.1)	1474 (63.0)	2
Slight/moderate dependence (Barthel index 61–99)	259 (4.6)	107 (4.0)	−3	161 (3.3)	73 (3.1)	−1
Independent (Barthel index = 100)	1402 (24.8)	612 (22.7)	−5	919 (18.8)	470 (20.1)	3
Missing	841 (14.9)	474 (17.6)	7	772 (15.8)	321 (13.7)	−6
Dementia before admission, n (%)	346 (6.1)	245 (9.1)	11	328 (6.7)	182 (7.8)	4
Home medical care before admission, n (%)	87 (1.5)	43 (1.6)	0	63 (1.3)	27 (1.2)	−1
Place before admission, n (%)						
Home	4858 (85.8)	2191 (81.4)	−12	4478 (91.5)	2096 (89.6)	−6
Other hospitals	776 (13.7)	479 (17.8)	11	366 (7.5)	221 (9.5)	7
Nursing home	25 (0.4)	22 (0.8)	5	48 (1.0)	21 (0.9)	−1
Ambulance use, *n* (%)	3674 (64.9)	1798 (66.8)	4	3632 (74.2)	1,704 (72.9)	−3
Primary diagnosis, *n* (%)						
Acute coronary syndrome	2068 (36.5)	796 (29.6)	−15	1770 (36.2)	843 (36.1)	0
Cardiac arrest	1032 (18.2)	411 (15.3)	−8	1284 (26.2)	643 (27.5)	3
Ventricular tachycardia or fibrillation	458 (8.1)	213 (7.9)	−1	446 (9.1)	188 (8.0)	−4
Heart failure	496 (8.8)	249 (9.2)	2	383 (7.8)	163 (7.0)	−3
Valve disease	597 (10.5)	294 (10.9)	1	236 (4.8)	108 (4.6)	−1
Myocarditis	215 (3.8)	80 (3.0)	−5	109 (2.2)	43 (1.8)	−3
Cardiomyopathy	203 (3.6)	94 (3.5)	−1	143 (2.9)	72 (3.1)	1
Aortic disease	530 (9.4)	256 (9.5)	0	318 (6.5)	140 (6.0)	−2
Extracorporeal cardiopulmonary resuscitation, n (%)	1888 (33.4)	736 (27.3)	−13	2283 (46.7)	1011 (43.2)	−7
Interventions before ECMO, n (%)						
Percutaneous coronary intervention	1744 (30.8)	684 (25.4)	−12	1536 (31.4)	756 (32.3)	2
Coronary artery bypass grafting	474 (8.4)	156 (5.8)	−10	360 (7.4)	183 (7.8)	2
Surgical valve procedures	295 (5.2)	124 (4.6)	−3	145 (3.0)	62 (2.7)	−2
Percutaneous valve procedures	262 (4.6)	155 (5.8)	5	71 (1.5)	32 (1.4)	−1
Organ failure supports on ECMO initiation, n (%)						
Red blood cell transfusion	3916 (69.2)	1836 (68.2)	−2	2867 (58.6)	1284 (54.9)	−7
Fresh frozen plasma transfusion	2931 (51.8)	1303 (48.4)	−7	2070 (42.3)	957 (40.9)	−3
Platelet transfusion	1524 (26.9)	689 (25.6)	−3	827 (16.9)	389 (16.6)	−1
Dopamine	2028 (35.8)	568 (21.1)	−33	1,676 (34.3)	560 (24.0)	−23
Dobutamine	2491 (44.0)	1071 (39.8)	−9	1678 (34.3)	823 (35.2)	2
Noradrenaline	4219 (74.6)	2072 (77.0)	6	3287 (67.2)	1689 (72.2)	11
Adrenaline	3547 (62.7)	1576 (58.5)	−8	3229 (66.0)	1524 (65.2)	−2
Vasopressin	618 (10.9)	318 (11.8)	3	320 (6.5)	230 (9.8)	12
Renal replacement therapy	1204 (21.3)	520 (19.3)	−5	894 (18.3)	369 (15.8)	−7
Anticoagulants on ECMO initiation, n (%)						
Heparin	5452 (96.3)	2577 (95.7)	−3	4654 (95.1)	2254 (96.4)	6
DOAC	94 (1.7)	62 (2.3)	5	68 (1.4)	44 (1.9)	3
Warfarin	152 (2.7)	69 (2.6)	−1	87 (1.8)	32 (1.4)	−3

*SMD* standardized mean difference, *ECMO* extracorporeal membrane oxygenation, *SD* standard deviation, *DOAC* direct oral anticoagulant

In-hospital mortality before and after exposure was 62.5% (3536/5659 patients) and 59.3% (1597/2692 patients), respectively, in the exposure group; and 66.8% (3266/4892 patients) and 63.7% (1489/2338 patients), respectively, in the control group (Table 2).

**Table 2:** Outcomes before and after the introduction of Impella in hospitals with and without Impella introduction

	Hospitals with Impella		Hospitals without Impella	
	Before	After	Before	After
	(*n* = 5659)	(*n* = 2692)	(*n* = 4892)	(*n* = 2338)
In-hospital mortality, *n* (%)	3536 (62.5)	1597 (59.3)	3266 (66.8)	1489 (63.7)
Length of hospital stay, days, mean (SD)	35.4 (55.3)	35.0 (50.2)	27.9 (45.4)	27.3 (39.6)
Length of ECMO, days, mean (SD)	4.2 (14.7)	5.9 (15.9)	3.2 (8.3)	3.5 (7.1)
Total hospitalization cost, × 10^3^ dollar, mean (SD)	63.4 (65.4)	73.4 (76.6)	46.5 (48.5)	47.2 (45.6)
Bleeding and ischemic complications, n (%)	307 (5.4)	126 (4.7)	187 (3.8)	97 (4.1)
Major bleeding, *n* (%)	128 (2.3)	52 (1.9)	83 (1.7)	34 (1.5)
Ischemic stroke, *n* (%)	190 (3.4)	76 (2.8)	105 (2.1)	64 (2.7)

*SD* standard deviation, *ECMO* extracorporeal membrane oxygenation

The uncontrolled interrupted time-series analysis showed a significant decreasing baseline trend of in-hospital mortality before exposure (−0.13%, 95% confidence intervals −0.23% to −0.03%), but no significant level change (+ 1.48%, −2.73% to + 5.69%) or trend change (+ 0.04%, −0.22% to + 0.31%) after exposure in the exposure group (Table 3). Similarly, there was a significant decreasing baseline trend of in-hospital mortality after Impella introduction (−0.11%, −0.19% to −0.03%), but no significant level change (+ 1.49%, −1.86% to + 4.85%) or trend change (−0.02%, −0.23% to + 0.19%) after exposure in the control group.

**Table 3:** Results of uncontrolled and controlled interrupted time series analyses

Outcome	Uncontrolled ITS				Controlled ITS	
	Hospitals	P	Hospitals	P		P
	With Impella	value	Without Impella	value	Difference	value
In-hospital mortality, %						
Baseline level	66.0 (63.2, 68.9)	–	69.9 (67.6, 72.3)	–	−3.89 (−7.56, −0.22)	0.038
Baseline trend	−0.13 (−0.23, −0.03)	0.012	−0.11 (−0.19, −0.03)	0.005	−0.02 (−0.14, 0.11)	0.772
Level change	1.48 (−2.73, 5.69)	0.487	1.49 (−1.86, 4.85)	0.379	−0.01 (−5.36, 5.33)	0.996
Trend change	0.04 (−0.22, 0.31)	0.751	−0.02 (−0.23, 0.19)	0.869	0.1 (−0.3, 0.4)	0.725
Length of hospital stay, days						
Baseline level	34.2 (30.3, 38.1)	–	27.8 (24.4, 31.2)	–	6.41 (1.28, 11.5)	0.015
Baseline trend	0.04 (−0.08, 0.17)	0.493	0.01 (−0.11, 0.12)	0.913	0.04 (−0.13, 0.20)	0.668
Level change	1.31 (−3.22, 5.84)	0.566	−0.99 (−5.47, 3.48)	0.659	2.31 (−4.01, 8.62)	0.472
Trend change	−0.25 (−0.47, −0.04)	0.020	0.00 (−0.22, 0.22)	0.981	−0.3 (−0.6, 0.1)	0.105
Length of ECMO, days						
Baseline level	3.1 (2.4, 3.8)	–	2.6 (2.2, 3,0)	–	0.49 (−0.32, 1.29)	0.233
Baseline trend	0.04 (0.02, 0.06)	< 0.001	0.02 (0.00, 0.04)	0.027	0.02 (−0.01, 0.05)	0.181
Level change	0.88 (−0.13, 1.89)	0.087	0.59 (−0.25, 1.43)	0.164	0.29 (−1.02, 1.59)	0.665
Trend change	−0.06 (−0.11, −0.01)	0.011	−0.08 (−0.11, −0.04)	< 0.001	0.0 (0.0, 0.1)	0.579
Hospitalization cost, × 10^3^ dollar						
Baseline level	59.4 (55.5, 63.4)	–	44.1 (40.6, 47.5)	–	15.4 (10.2, 20.6)	< 0.001
Baseline trend	0.13 (0.02, 0.25)	0.025	0.09 (−0.02, 0.20)	0.117	0.04 (−0.12, 0.20)	0.600
Level change	6.43 (0.34, 12.5)	0.039	−0.76 (−5.36, 3.84)	0.742	7.19 (−0.38, 14.8)	0.062
Trend change	−0.07 (−0.36, 0.22)	0.639	−0.16 (−0.39, 0.07)	0.160	0.09 (−0.28, 0.46)	0.622
Complications, %						
Baseline level	5 (3.7, 6.4)	–	3.9 (2.5, 5.3)	–	1.10 (−0.83, 3.03)	0.262
Baseline trend	0.01 (−0.03, 0.06)	0.527	0.00 (−0.05, 0.04)	0.942	0.02 (−0.05, 0.08)	0.612
Level change	−0.87 (−3.10, 1.36)	0.438	0.99 (−1.25, 3.24)	0.382	−1.86 (−5.00, 1.27)	0.242
Trend change	−0.03 (−0.15, 0.09)	0.651	−0.06 (−0.18, 0.06)	0.350	0.0 (−0.1, 0.2)	0.739
Major bleeding, %						
Baseline level	1.8 (1.0, 2.6)	–	1.6 (0.8, 2.4)	–	0.20 (−0.96, 1.36)	0.735
Baseline trend	0.02 (−0.01, 0.04)	0.270	0.00 (−0.02, 0.03)	0.812	0.01 (−0.03, 0.05)	0.512
Level change	−0.16 (−1.38, 1.06)	0.796	0.64 (−1.06, 2.34)	0.457	−0.80 (−2.87, 1.28)	0.449
Trend change	−0.05 (−0.12, 0.02)	0.184	−0.07 (−0.16, 0.01)	0.090	0.0 (−0.1, 0.1)	0.663
Ischemic stroke, %						
Baseline level	3.4 (2.4, 4.4)	–	2.4 (1.5, 3.3)	–	1.04 (−0.28, 2.35)	0.121
Baseline trend	0.00 (−0.03, 0.03)	0.988	−0.01 (−0.03, 0.02)	0.707	0.01 (−0.04, 0.05)	0.800
Level change	−0.94 (−2.47, 0.59)	0.226	0.39 (−1.10, 1.87)	0.605	−1.32 (−3.44, 0.79)	0.218
Trend change	0.02 (−0.04, 0.09)	0.479	0.02 (−0.08, 0.11)	0.704	0.0 (−0.1, 0.1)	0.904

*ITS* interrupted time series analysis, *ECMO* extracorporeal membrane oxygenation

**Fig. 2:**
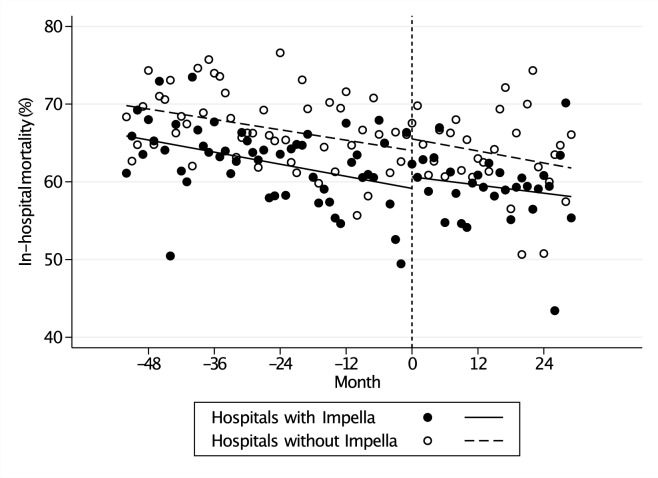
Trends of in-hospital mortality in exposure group (hospitals with Impella introduction) and control group (hospitals without Impella introduction) before and after exposure. Closed circles and black regression line denote exposure group. Open circles and dashed regression line denote control group. Vertical dashed line indicates the time of Impella introduction. Impella, percutaneous ventricular assist device

The controlled interrupted time-series analysis showed a significant baseline level difference in in-hospital mortality at the beginning of the study period between the exposure and control groups (−3.89%, −7.56% to −0.22%), but no significant baseline trend difference before Impella introduction in the exposure–control comparison (-0.02%, −0.14% to + 0.11%) (Table 3 and Fig. 2). After Impella introduction, there was no significant level change (−0.01%, −5.36% to + 5.33%) and no trend change (+ 0.10%, −0.30% to + 0.40%) after Impella introduction in the exposure–control comparison.

The controlled interrupted time-series analysis showed a significant baseline level difference in length of hospital stay (6.41 days, 1.28 to 11.5 days) and hospitalization costs (15.4 thousand dollars, 10.2 to 20.6 thousand dollars) at the beginning of the study period between the exposure and control groups. As for the other secondary outcomes, the controlled interrupted time-series analysis showed no significant baseline level or trend change at the beginning of the study period and level or trend change after Impella introduction in the exposure–control comparisons.

## Discussion

To the best of our knowledge, this is the first report on the impact of introduction of Impella in hospitals on in-hospital mortality of patients treated with ECMO. The results showed that Impella introduction was not associated with reduced mortality, complication rates, length of hospital stay, duration of ECMO, or total hospitalization costs at the hospital level.

The baseline in-hospital mortality among patients treated with ECMO in hospitals that introduced Impella was lower than the mortality in hospitals that did not introduce Impella. This finding may reflect the fact that hospitals implementing Impella are certified facilities with sufficiently high capacity and quality to manage a large volume of t-MCS. However, the controlled interrupted time series analysis showed that hospitals with and those without Impella introduction did not differ significantly in the level or trend of in-hospital mortality in ECMO-treated patients after Impella introduction in hospitals. Although randomized controlled trials on LV unloading strategies are certainly needed to reach a conclusion about the benefits of using Impella with ECMO, several patient-level reports have proposed the beneficial outcome of using Impella in patients undergoing ECMO. A meta-analysis comparing ECMO patients with and without Impella showed that patients with ECMO and Impella had a lower likelihood of short-term mortality and a higher likelihood of progression to durable left ventricular assist device or heart transplant than patients with ECMO alone [20]. The reason for the discrepancy of our result with others may be due to the low rate of Impella use (14.3%, 374 of 2692 patients treated with ECMO) in the exposure group after Impella introduction. In addition, since the study covered the first 30 months after Impella was introduced, there was a possibility that the Impella technique was not yet mature enough to provide benefit due to learning curve issues [21]. Furthermore, in interpreting the present results, it is important to note that this study did not examine the effect of using Impella on individuals, but rather investigated whether the introduction of Impella in hospitals would benefit the ECMO patient population in the hospitals.

Meanwhile, the total hospitalization costs tended to be higher in the Impella introduction group than in the non-Impella introduction group, although the difference was not statistically significant. This may be primarily because of the significantly higher baseline inpatient costs in the Impella introduction group compared to the non-Impella introduction group even before Impella was introduced. Another factor may be the considerably higher cost of Impella compared to ECMO or IABP. According to Japanese insurance reimbursement data, the total hospitalization costs of the Impella group were approximately 1.5 times higher than the ECMO group and 2 times higher than the IABP group [22].

Based on the result of our analysis, new facilities planning to introduce Impella should consider the balance of the anticipated clinical benefit and the increasing costs for facility and staffing structure and implementation. This study aimed to analyse the hospital-level impact of the introduction of Impella on patients who underwent ECMO, and excluded patients supported with Impella alone.

Recently, a multicenter randomized study (the DanGer Shock trial) showed that all-cause mortality within 180 days was significantly lower in the Impella CP group than in the standard care group for the patients with STEMI complicated by cardiogenic shock (45.8% versus 58.5%; HR 0.74, 95% CI 0.55–0.99, P = 0.04). In addition, according to the Japan Registry for Percutaneous Ventricular Assist Device (J-PVAD), survival of patients supported only with Impella was higher than patients supported with Impella combined with venoarterial ECMO (81.1% vs 49.6%) [23]. The introduction of Impella should be considered based on the total effect of different types of Impella support in the entire target population. Hence, our results should be interpreted carefully. In addition, establishing a system for proactive and appropriate management using Impella concomitant with ECMO is warranted. Recent guidelines recommend that developing a regional care system integrating t-MCS–capable hub hospitals and spoke centres together with defined protocols for early recognition, treatment, and transfer may improve the outcomes of cardiogenic shock patients [8].

The present study had some limitations. First, this study did not examine the effect of Impella introduction in hospitals on the clinical outcome of patients supported with Impella alone. In addition, we did not examine the effect of initiation timing, support flow, and the types of Impella on heart recovery associated with LV unloading by Impella at the patient level. Second, the observational period was the first 2.5 years of introducing Impella in hospitals. The short, early period of implementing a new technology may have contributed to the limited clinical effect observed in this study. Third, in the controlled interrupted time-series analysis, the comparison between the exposure and control groups was not corrected for the model, which may have resulted in unbalanced hospital attributes. Fourth, the implementation cost of Impella was not included in the total hospitalization costs. Thus, additional long-term analysis is needed to conclude the real impact of introducing Impella at the hospital level.

## Conclusions

This study using a nationwide inpatient administrative database showed that introducing Impella in hospitals was not associated with beneficial effects for ECMO-treated patients in the short, early period of Impella introduction. Because this study confined itself to analyze of the impact of the introduction of Impella solely at the hospital level, further detailed studies are warranted to assess its efficacy at the patient level.

## Supplementary Information

Additional file 1.

## Data Availability

Data sharing is not applicable to this article as no new data sets were generated or analysed during the current study.

## Abbreviations

ECMO: Extracorporeal membrane oxygenation
IABP: Intra-aortic balloon pumping
LV: Left ventricular
PCPS: Percutaneous cardio-pulmonary support
t-MCS: Temporary mechanical circulatory support
