# Supplementary material for: The association between introduction of the micro-axial flow pump Impella in hospitals and in-hospital mortality in patients treated with extracorporeal membrane oxygenation: interrupted time-series analyses
Source: Ann Intensive Care. 2024 Sep 28;14:151. doi: 10.1186/s13613-024-01381-4 (PMC11438750; doi:10.1186/s13613-024-01381-4)
Supplement: Supplementary file 1 — Additional file 1. [file 13613_2024_1381_MOESM1_ESM.docx]

***Supplementary Materials***

**The association between introduction of the micro-axial flow pump Impella in hospitals and in-hospital mortality in patients treated with extracorporeal membrane oxygenation: Interrupted time-series analyses**

**Authors: Jun Nakata^1^, Hiroyuki Ohbe^2*^, Toru Takiguchi^3^, Yuji Nishimoto^4^, Mikio Nakajima^2,5^, Yusuke Sasabuchi^6^, Toshiaki Isogai^2,7^, Hiroki Matsui^2^, Takeshi Yamamoto^1^, Shoji Yokobori^3^, Kuniya Asai^1^ and Hideo Yasunaga^2^**

**Affiliations: ^1^Division of Cardiovascular Intensive Care, Nippon Medical School, Tokyo, Japan. ^2^Department of Clinical Epidemiology and Health Economics, School of Public Health, The University of Tokyo, Tokyo, Japan. ^3^Department of Emergency and Critical Care Medicine, Nippon Medical School, Tokyo, Japan. ^4^Division of Cardiology, Osaka General Medical Center, Osaka, Japan. ^5^Emergency and Critical Care Center, Tokyo Metropolitan Hiroo Hospital, Tokyo, Japan. ^6^Department of Real-world Evidence, Graduate School of Medicine, The University of Tokyo, Tokyo, Japan. ^7^Department of Cardiology, Tokyo Metropolitan Tama Medical Center, Tokyo, Japan.**

***Correspondence: Hiroyuki Ohbe**

**Department of Clinical Epidemiology and Health Economics, School of Public Health, The University of Tokyo, 7-3-1 Hongo, Bunkyo-ku, Tokyo 1130033, Japan**

**TEL: +81-3-5841-1887 FAX: +81-3-5841-1888 E-mail:** [**hohbey@gmail.com**](mailto:hohbey@gmail.com)

**Supplement Table 1**. **ICD-10 codes for cardiovascular diseases**

| Primary diagnosis | ICD-10 codes |
| --- | --- |
| Acute coronary syndrome | I20 I21 I22 I23 I24 |
| Aortic diseases | I71 |
| Valve diseases | I05 I06 I07 I08 I09 I34 I35 I36 I37 |
| Heart failure | I50 |
| Cardiac arrest | I46 |
| Ventricular tachycardia or fibrillation | I472 I490 |
| Pulmonary embolism | I26 |
| Myocarditis | I40 |
| Cardiomyopathy | I25 I42 |

**Supplement Table 2**. Hospital characteristics before and after hospital-level propensity score matching.

|  | Before hospital-level PS matching | |  | After hospital-level PS matching | |
| --- | --- | --- | --- | --- | --- |
|  | Hospitals | Hospitals |  | Hospitals | Hospitals |
|  | with Impella | without Impella |  | with Impella | without Impella |
| Hospital characteristics | (*n* = 129) | (*n* = 209) |  | (*n* = 86) | (*n* = 86) |
| Teaching hospital, n (%) | 127 (98.4) | 205 (98.1) |  | 85 (98.8) | 86 (100.0) |
| Tertiary emergency hospital, n (%) | 91 (70.5) | 95 (45.5) |  | 56 (65.1) | 63 (73.3) |
| Annual hospital volume of ECMO, mean (SD) | 52.5 (27.2) | 25.8 (17.8) |  | 47.5 (27.8) | 34.2 (20.8) |

PS, propensity score; ECMO, extracorporeal membrane oxygenation; SD, standard deviation.

**Supplement Table 3**. Patient characteristics between hospitals with and without Impella introduction.

|  | Hospitals | Hospitals |  |
| --- | --- | --- | --- |
|  | with Impella | without Impella | |
|  | (*n* = 8,351) | (*n* = 7,230) | SMD |
| Fiscal year at admission, n (%) |  |  |  |
| 2014 | 297 (3.6) | 296 (4.1) | -3 |
| 2015 | 716 (8.6) | 573 (7.9) | 2 |
| 2016 | 1,053 (12.6) | 907 (12.5) | 0 |
| 2017 | 1,388 (16.6) | 1,084 (15.0) | 4 |
| 2018 | 1,351 (16.2) | 1,222 (16.9) | -2 |
| 2019 | 1,382 (16.5) | 1,229 (17.0) | -1 |
| 2020 | 1,194 (14.3) | 1,123 (15.5) | -3 |
| 2021 | 970 (11.6) | 796 (11.0) | 2 |
| Hospital characteristics |  |  |  |
| Teaching hospital, n (%) | 8,309 (99.5) | 7,230 (100.0) | -10 |
| Tertiary emergency hospital, n (%) | 6,062 (72.6) | 5,967 (82.5) | -24 |
| Annual hospital volume of ECMO, mean (SD) | 61.5 (26.9) | 44.9 (20.7) | 69 |
| Age, years, mean (SD) | 64.4 (15.5) | 64.5 (14.5) | -1 |
| Men, *n* (%) | 5,829 (69.8) | 5,283 (73.1) | -7 |
| Smoking history, n (%) |  |  |  |
| Nonsmoker | 3,483 (41.7) | 3,077 (42.6) | -2 |
| Current/past smoker | 2,826 (33.8) | 2,260 (31.3) | 6 |
| Unknown | 2,042 (24.5) | 1,893 (26.2) | -4 |
| Body mass index at admission, kg/m^2^, n (%) |  |  |  |
| <18.5 | 719 (8.6) | 472 (6.5) | 8 |
| 18.5–24.9 | 4,034 (48.3) | 3,297 (45.6) | 5 |
| 25.0–29.9 | 1,814 (21.7) | 1,549 (21.4) | 1 |
| ≥30.0 | 656 (7.9) | 560 (7.7) | 0 |
| Missing data | 1,128 (13.5) | 1,352 (18.7) | -14 |
| Japan Coma Scale at admission, *n* (%) |  |  |  |
| 0 (alert) | 4,599 (55.1) | 3,019 (41.8) | 27 |
| 1-3 (dizzy) | 737 (8.8) | 560 (7.7) | 4 |
| 10-30 (somnolent) | 367 (4.4) | 278 (3.8) | 3 |
| 100-300 (coma) | 2,648 (31.7) | 3,373 (46.7) | -31 |
| Charlson comorbidity index score, mean (SD) | 1.1 (1.3) | 1.0 (1.3) | 8 |
| Comorbidity of peripheral vascular diseases, n (%) | 420 (5.0) | 265 (3.7) | 7 |
| Physical function at admission, n (%) |  |  |  |
| Total/severe dependence (Barthel index 0-60) | 4,656 (55.8) | 4,514 (62.4) | -14 |
| Slight/moderate dependence (Barthel index 61-99) | 366 (4.4) | 234 (3.2) | 6 |
| Independent (Barthel index = 100) | 2,014 (24.1) | 1,389 (19.2) | 12 |
| Missing | 1,315 (15.7) | 1,093 (15.1) | 2 |
| Dementia before admission, n (%) | 591 (7.1) | 510 (7.1) | 0 |
| Home medical care before admission, n (%) | 130 (1.6) | 90 (1.2) | 3 |
| Place before admission, n (%) |  |  |  |
| Home | 7,049 (84.4) | 6,574 (90.9) | -20 |
| Other hospitals | 1,255 (15.0) | 587 (8.1) | 22 |
| Nursing home | 47 (0.6) | 69 (1.0) | -5 |
| Ambulance use, *n* (%) | 5,472 (65.5) | 5,336 (73.8) | -18 |
| Primary diagnosis, *n* (%) |  |  |  |
| Acute coronary syndrome | 2,864 (34.3) | 2,613 (36.1) | -4 |
| Cardiac arrest | 1,443 (17.3) | 1,927 (26.7) | -23 |
| Ventricular tachycardia or fibrillation | 671 (8.0) | 634 (8.8) | -3 |
| Heart failure | 745 (8.9) | 546 (7.6) | 5 |
| Valve disease | 891 (10.7) | 344 (4.8) | 22 |
| Myocarditis | 295 (3.5) | 152 (2.1) | 9 |
| Cardiomyopathy | 297 (3.6) | 215 (3.0) | 3 |
| Aortic disease | 786 (9.4) | 458 (6.3) | 11 |
| Extracorporeal cardiopulmonary resuscitation, n (%) | 2,624 (31.4) | 3,294 (45.6) | -29 |
| Interventions before ECMO, n (%) |  |  |  |
| Percutaneous coronary intervention | 2,428 (29.1) | 2,292 (31.7) | -6 |
| Coronary artery bypass grafting | 630 (7.5) | 543 (7.5) | 0 |
| Surgical valve procedures | 419 (5.0) | 207 (2.9) | 11 |
| Percutaneous valve procedures | 417 (5.0) | 103 (1.4) | 20 |
| Organ failure supports on ECMO initiation, n (%) | |  |  |
| Red blood cell transfusion | 5,752 (68.9) | 4,151 (57.4) | 24 |
| Fresh frozen plasma transfusion | 4,234 (50.7) | 3,027 (41.9) | 18 |
| Platelet transfusion | 2,213 (26.5) | 1,216 (16.8) | 24 |
| Dopamine | 2,596 (31.1) | 2,236 (30.9) | 0 |
| Dobutamine | 3,562 (42.7) | 2,501 (34.6) | 17 |
| Noradrenaline | 6,291 (75.3) | 4,976 (68.8) | 15 |
| Adrenaline | 5,123 (61.3) | 4,753 (65.7) | -9 |
| Vasopressin | 936 (11.2) | 550 (7.6) | 12 |
| Renal replacement therapy | 1,724 (20.6) | 1,263 (17.5) | 8 |
| Anticoagulants on ECMO initiation, n (%) |  |  |  |
| Heparin | 8.029 (96.1) | 6.907 (95.5) | 3 |
| DOAC | 156 (1.9) | 112 (1.5) | 3 |
| Warfarin | 221 (2.6) | 119 (1.6) | 7 |

SMD, standardized mean difference; ECMO, extracorporeal membrane oxygenation; SD, standard deviation; DOAC, direct oral anticoagulant.

**Supplement Table 4**. Outcomes between hospitals with and without Impella introduction.

|  | Hospitals | Hospitals |
| --- | --- | --- |
|  | with Impella | without Impella |
|  | (*n* = 8,351) | (*n* = 7,230) |
| In-hospital mortality, *n* (%) | 5,133 (61.5) | 4,755 (65.8) |
| Length of hospital stay, days, mean (SD) | 35.3 (53.7) | 27.7 (43.6) |
| Length of ECMO, days, mean (SD) | 4.7 (15.1) | 3.3 (7.9) |
| Total hospitalization cost, ×10^3^ dollar, mean (SD) | 66.6 (69.4) | 46.7 (47.6) |
| Bleeding and ischemic complications, n (%) | 433 (5.2) | 284 (3.9) |
| Major bleeding, *n* (%) | 180 (2.2) | 117 (1.6) |
| Ischemic stroke, *n* (%) | 266 (3.2) | 169 (2.3) |

SD, standard deviation; ECMO, extracorporeal membrane oxygenation.

**Supplement Table 5**. Patient characteristics and outcomes between patients receiving ECMO with and without Impella in hospitals with Impella after exposure

|  | ECMO | ECMO |  |
| --- | --- | --- | --- |
|  | with Impella | without Impella | |
|  | (*n* = 384) | (*n* = 2,318) | SMD |
| Fiscal year at admission, n (%) |  |  |  |
| 2017 | 7 (1.8) | 13 (0.6) | 12 |
| 2018 | 36 (9.4) | 162 (7.0) | 10 |
| 2019 | 110 (28.6) | 585 (25.2) | 9 |
| 2020 | 108 (28.1) | 781 (33.7) | -10 |
| 2021 | 113 (29.4) | 777 (33.5) | -7 |
| Hospital characteristics |  |  |  |
| Teaching hospital, n (%) | 373 (97.1) | 2,311 (99.7) | 1 |
| Tertiary emergency hospital, n (%) | 258 (67.2) | 1,657 (71.5) | -6 |
| Annual hospital volume of ECMO, mean (SD) | 59.7 (24.8) | 63.9 (24.2) | -17 |
| Age, years, mean (SD) | 62.5 (15.0) | 64.7 (15.5) | -15 |
| Men, *n* (%) | 275 (71.6) | 1,569 (67.7) | 13 |
| Smoking history, n (%) |  |  |  |
| Nonsmoker | 155 (40.4) | 978 (42.2) | -2 |
| Current/past smoker | 140 (36.5) | 725 (31.3) | 13 |
| Unknown | 79 (20.6) | 615 (26.5) | -13 |
| Body mass index at admission, kg/m^2^, n (%) |  |  |  |
| <18.5 | 29 (7.6) | 197 (8.5) | -3 |
| 18.5–24.9 | 188 (49.0) | 1,055 (45.5) | 10 |
| 25.0–29.9 | 85 (22.1) | 514 (22.2) | 1 |
| ≥30.0 | 39 (10.2) | 213 (9.2) | 4 |
| Missing data | 33 (8.6) | 339 (14.6) | -18 |
| Japan Coma Scale at admission, *n* (%) |  |  |  |
| 0 (alert) | 202 (52.6) | 1,277 (55.1) | -2 |
| 1-3 (dizzy) | 43 (11.2) | 213 (9.2) | 8 |
| 10-30 (somnolent) | 24 (6.3) | 105 (4.5) | 8 |
| 100-300 (coma) | 105 (27.3) | 723 (31.2) | -7 |
| Charlson comorbidity index score, mean (SD) | 1.2 (1.2) | 1.1 (1.4) | 7 |
| Comorbidity of peripheral vascular diseases, n (%) | 13 (3.4) | 114 (4.9) | -7 |
| Physical function at admission, n (%) |  |  |  |
| Total/severe dependence (Barthel index 0-60) | 234 (60.9) | 1,265 (54.6) | 16 |
| Slight/moderate dependence (Barthel index 61-99) | 12 (3.1) | 95 (4.1) | -5 |
| Independent (Barthel index = 100) | 59 (15.4) | 553 (23.9) | -20 |
| Missing | 69 (18.0) | 405 (17.5) | 4 |
| Dementia before admission, n (%) | 26 (6.8) | 219 (9.4) | -9 |
| Home medical care before admission, n (%) | 4 (1.0) | 39 (1.7) | -5 |
| Place before admission, n (%) |  |  |  |
| Home | 18 (4.7) | 397 (17.1) | -13 |
| Other hospitals | 82 (21.4) | 1,903 (82.1) | 12 |
| Nursing home | 4 (1.0) | 288 (12.4) | 3 |
| Ambulance use, *n* (%) | 283 (73.7) | 1,515 (65.4) | 23 |
| Primary diagnosis, *n* (%) |  |  |  |
| Acute coronary syndrome | 198 (51.6) | 598 (25.8) | 58 |
| Cardiac arrest | 52 (13.5) | 359 (15.5) | -5 |
| Ventricular tachycardia or fibrillation | 20 (5.2) | 193 (8.3) | -12 |
| Heart failure | 63 (16.4) | 186 (8.0) | 27 |
| Valve disease | 25 (6.5) | 269 (11.6) | -17 |
| Myocarditis | 52 (13.5) | 28 (1.2) | 49 |
| Cardiomyopathy | 26 (6.8) | 68 (2.9) | 19 |
| Aortic disease | 11 (2.9) | 245 (10.6) | -31 |
| Extracorporeal cardiopulmonary resuscitation, n (%) | 64 (16.7) | 672 (29.0) | -29 |
| Interventions before ECMO, n (%) |  |  |  |
| Percutaneous coronary intervention | 188 (49.0) | 496 (21.4) | 63 |
| Coronary artery bypass grafting | 25 (6.5) | 131 (5.7) | 4 |
| Surgical valve procedures | 25 (6.5) | 99 (4.3) | 11 |
| Percutaneous valve procedures | 3 (0.8) | 152 (6.6) | -31 |
| Organ failure supports on ECMO initiation, n (%) |  |  |  |
| Red blood cell transfusion | 298 (77.6) | 1,538 (66.4) | 30 |
| Fresh frozen plasma transfusion | 227 (59.1) | 1,076 (46.4) | 29 |
| Platelet transfusion | 122 (31.8) | 567 (24.5) | 18 |
| Dopamine | 86 (22.4) | 482 (20.8) | 5 |
| Dobutamine | 200 (52.1) | 871 (37.6) | 32 |
| Noradrenaline | 303 (78.9) | 1.769 (76.3) | 12 |
| Adrenaline | 195 (50.8) | 1,381 (59.6) | -15 |
| Vasopressin | 39 (10.2) | 279 (12.0) | -5 |
| Renal replacement therapy | 96 (25) | 424 (18.3) | 18 |
| Anticoagulants on ECMO initiation, n (%) |  |  |  |
| Heparin | 366 (95.3) | 2,211 (95.4) | 14 |
| DOAC | 3 (0.8) | 59 (2.5) | -14 |
| Warfarin | 7 (1.8) | 62 (2.7) | -5 |
| Outcomes |  |  |  |
| In-hospital mortality, *n* (%) | 238 (62.0) | 1,359 (58.6) |  |
| Length of hospital stay, days, mean (SD) | 48.7 (77.7) | 32.7 (43.8) |  |
| Length of ECMO, days, mean (SD) | 7.0 (26.5) | 5.7 (13.4) |  |
| Total hospitalization cost, ×10^3^ dollar, mean (SD) | 124.1 (108.6) | 65.2 (66.6) |  |
| Bleeding and ischemic complications, n (%) | 22 (5.7) | 104 (4.5) |  |
| Major bleeding, *n* (%) | 12 (3.1) | 40 (1.7) |  |
| Ischemic stroke, *n* (%) | 10 (2.6) | 66 (2.8) |  |

SMD, standardized mean difference; ECMO, extracorporeal membrane oxygenation; SD, standard deviation; DOAC, direct oral anticoagulant.
